# *catena*-Poly[[aqua­(pyrazino[2,3-*f*][1,10]phenanthroline-κ^2^ *N* ^8^,*N* ^9^)cobalt(II)]-μ-pyrazine-2,3-dicarboxyl­ato-κ^3^ *N* ^1^ *O* ^2^:*O* ^3^]

**DOI:** 10.1107/S1600536808027177

**Published:** 2008-08-30

**Authors:** Zhan-Lin Xu, Xiu-Ying Li, Guang-Bo Che, Lu Lu, Chun-Hui Xu

**Affiliations:** a Department of Chemistry, Jilin Normal University, Siping 136000, People’s Republic of China

## Abstract

In the title compound, [Co(C_6_H_2_N_2_O_4_)(C_14_H_8_N_4_)(H_2_O)]_*n*_, the Co atom is bonded to one *N*,*N*′-bidentate pyrazino[2,3-*f*][1,10]phenanthroline (Pyphen) ligand, one *N*,*O*-bidentate pyrazine-2,3-dicarboxyl­ate (PZDC) dianion and one water mol­ecule in a distorted octa­hedral *mer*-CoN_3_O_3_ geometry. The Co^II^ atoms are bridged by the PZDC dianions, forming an infinite one-dimensional chain running along the *b* axis. Adjacent chains pack together through π–π stacking inter­actions [centroid–centroid separations = 3.498 (4) and 3.528 (4) Å], and O—H⋯O and O—H⋯N hydrogen bonds involving the water mol­ecule complete the structure.

## Related literature

For related structures, see: Che *et al.* (2008 ▶); Liu *et al.* (2008 ▶). For the synthesis of the ligand, see: Che *et al.* (2006 ▶).
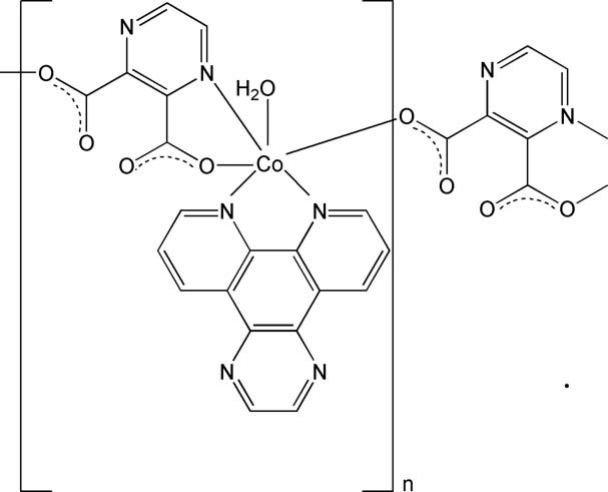


## Experimental

### 

#### Crystal data

[Co(C_6_H_2_N_2_O_4_)(C_14_H_8_N_4_)(H_2_O)]
*M* *_r_* = 475.29
Triclinic, 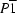

*a* = 6.8430 (14) Å
*b* = 7.4455 (15) Å
*c* = 17.454 (4) Å
α = 93.64 (3)°
β = 95.99 (3)°
γ = 97.61 (3)°
*V* = 873.9 (3) Å^3^
*Z* = 2
Mo *K*α radiation
μ = 1.04 mm^−1^
*T* = 292 (2) K
0.41 × 0.33 × 0.19 mm

#### Data collection

Bruker SMART CCD diffractometer
Absorption correction: multi-scan (*SADABS*; Bruker, 2002 ▶) *T*_min_ = 0.672, *T*_max_ = 0.823
7576 measured reflections
3434 independent reflections
1541 reflections with *I* > 2σ(*I*)
*R*_int_ = 0.114

#### Refinement

*R*[*F* ^2^ > 2σ(*F* ^2^)] = 0.070
*wR*(*F* ^2^) = 0.161
*S* = 0.92
3434 reflections
297 parameters
H atoms treated by a mixture of independent and constrained refinement
Δρ_max_ = 0.45 e Å^−3^
Δρ_min_ = −0.65 e Å^−3^

### 

Data collection: *SMART* (Bruker, 2002 ▶); cell refinement: *SAINT* (Bruker, 2002 ▶); data reduction: *SAINT*; program(s) used to solve structure: *SHELXS97* (Sheldrick, 2008 ▶); program(s) used to refine structure: *SHELXL97* (Sheldrick, 2008 ▶); molecular graphics: *SHELXTL* (Sheldrick, 2008 ▶); software used to prepare material for publication: *SHELXTL*.

## Supplementary Material

Crystal structure: contains datablocks global, I. DOI: 10.1107/S1600536808027177/hb2775sup1.cif

Structure factors: contains datablocks I. DOI: 10.1107/S1600536808027177/hb2775Isup2.hkl

Additional supplementary materials:  crystallographic information; 3D view; checkCIF report

## Figures and Tables

**Table 1:** Selected bond lengths (Å)

Co—N1	2.116 (5)
Co—N2	2.124 (5)
Co—N5	2.135 (5)
Co—O3^i^	2.050 (5)
Co—O1*W*	2.110 (5)
Co—O1	2.125 (5)

Symmetry code: (i) 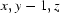.

**Table 2:** Hydrogen-bond geometry (Å, °)

*D*—H⋯*A*	*D*—H	H⋯*A*	*D*⋯*A*	*D*—H⋯*A*
O1*W*—H*W*1*A*⋯O4^ii^	0.95 (7)	1.76 (7)	2.680 (7)	164 (6)
O1*W*—H*W*1*B*⋯N6^iii^	0.78 (7)	2.15 (7)	2.851 (8)	149 (7)

Symmetry codes: (ii) 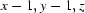; (iii) 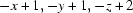.

**Fig. 1.**
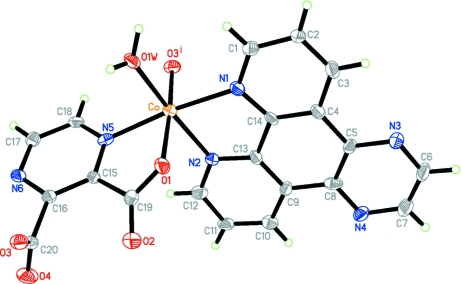
The asymmetric unit of (I), expanded to show the metal coordination sphere. Displacement ellipsoids are drawn at the 30% probability level (arbitrary spheres for the H atoms). [Symmetry code: (i) x, y - 1, z.]

**Fig. 2.**
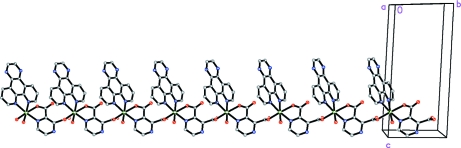
View of one-dimensional chain structure of (I). H atoms have been omitted.

**Fig. 3.**
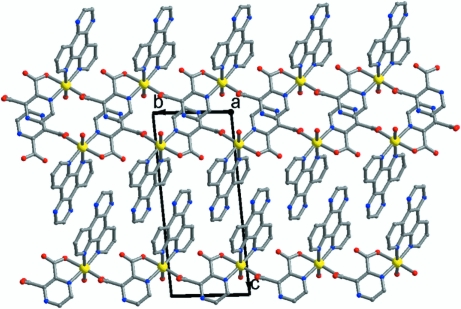
View of three-dimensional superamolecular structure of (I) built up viaπ-π interactions. H atoms have been omitted.

## supplementary crystallographic information

### Comment

As part of our ongoing studies of supramolecular architectures containing pyrazino[2,3-*f*][1,10]phenanthroline (Pyphen) (Che, Liu *et al.*, 2008; Liu *et al.*, 2008), we selected pyrazine-2,3-dicarboxylic acid (H_2_PZDC) as a linker and Pyphen as a secondary ligandin combination with Co^2+^ ions, forming the title compound, (I), a new coordination polymer, which is reported here.

In compound (I), each Co atom is six-coordinated by three N atoms from one Pyphen ligand and one PZDC^2-^ ligand, and three O atoms from two PZDC^2-^ ligands and one water molecule in a slightly distorted octahedral geometry (Fig. 1) with O1W, N1, N2 and N5 forming the equatorial plane, and O1 and O3 in the axial positions (Table 1). One carboxylate oxygen atom and pyrazine nitrogen atom of PZDC^2-^ chelate one Co(II) ion, while the other carboxylate oxygen atom is coordinated to another Co(II) ion in a monodentate fashion, forming an infinite one-dimensional chain running along the *b* axis (Fig. 2).

Adjacent chains pack together through π-π stacking interactions between the Pyphen ligands at a centroid separation of 3.498 (4) Å and between the PZDC^2-^ ligands from adjacent one-dimensional chains at centroid separation 3.528 (4)Å, resulting in a three-dimensional supramolecular structure (Fig. 3).

Finally, O—H···O and O—H···N hydrogen bonds involving the water molecules and the O4 and N6 atoms of the PZDC^2-^ dianion acceptors complete the structure of (I) (Table 2).

### Experimental

The Pyphen ligand was synthesized according to the literature method (Che *et al.*,  2006).  A mixture of Pyphen, H_2_PZDC, Co(NO_3_)_2_ and water in a molar ratio of 1:1:1:5000 was sealed in a Teflon-lined autoclave and heated to 433 K for 3 d. Upon cooling and opening the bomb, yellow blocks of (I) were obtained (76% yield based on Co).

### Refinement

All H atoms on C atoms were positioned geometrically (C—H = 0.93 Å) and refined as riding, with *U*_iso_(H)= 1.2*U*_eq_(C). The hydrogen atoms of water molecules were located from difference Fourier maps and their positions and *U*_iso_ values were refined freely.

### Crystal data

**Table:**

[Co(C_6_H_2_N_2_O_4_)(C_14_H_8_N_4_)(H_2_O)]	*Z* = 2
*M**_r_* = 475.29	*F*_000_ = 482
Triclinic, *P*1	*D*_x_ = 1.806 Mg m^−^^3^
Hall symbol: -P 1	Mo *K*α radiation λ = 0.71073 Å
*a* = 6.8430 (14) Å	Cell parameters from 2411 reflections
*b* = 7.4455 (15) Å	θ = 2.4–26.0º
*c* = 17.454 (4) Å	µ = 1.04 mm^−^^1^
α = 93.64 (3)º	*T* = 292 (2) K
β = 95.99 (3)º	Block, yellow
γ = 97.61 (3)º	0.41 × 0.33 × 0.19 mm
*V* = 873.9 (3) Å^3^	

### Data collection

**Table:**

Bruker SMART CCD diffractometer	3434 independent reflections
Radiation source: fine-focus sealed tube	1541 reflections with *I* > 2σ(*I*)
Monochromator: graphite	*R*_int_ = 0.114
*T* = 292(2) K	θ_max_ = 26.1º
ω scans	θ_min_ = 2.4º
Absorption correction: multi-scan(SADABS; Bruker, 2002)	*h* = −8→8
*T*_min_ = 0.672, *T*_max_ = 0.823	*k* = −9→9
7576 measured reflections	*l* = −20→21

### Refinement

**Table:**

Refinement on *F*^2^	Secondary atom site location: difference Fourier map
Least-squares matrix: full	Hydrogen site location: difmap and geom
*R*[*F*^2^ > 2σ(*F*^2^)] = 0.070	H atoms treated by a mixture of independent and constrained refinement
*wR*(*F*^2^) = 0.161	*w* = 1/[σ^2^(*F*_o_^2^) + (0.0488*P*)^2^] where *P* = (*F*_o_^2^ + 2*F*_c_^2^)/3
*S* = 0.92	(Δ/σ)_max_ < 0.001
3434 reflections	Δρ_max_ = 0.45 e Å^−^^3^
297 parameters	Δρ_min_ = −0.65 e Å^−^^3^
Primary atom site location: structure-invariant direct methods	Extinction correction: none

### Special details

**Table:**

Geometry. All e.s.d.'s (except the e.s.d. in the dihedral angle between two l.s. planes) are estimated using the full covariance matrix. The cell e.s.d.'s are taken into account individually in the estimation of e.s.d.'s in distances, angles and torsion angles; correlations between e.s.d.'s in cell parameters are only used when they are defined by crystal symmetry. An approximate (isotropic) treatment of cell e.s.d.'s is used for estimating e.s.d.'s involving l.s. planes.
Refinement. Refinement of *F*^2^ against ALL reflections. The weighted *R*-factor *wR* and goodness of fit *S* are based on *F*^2^, conventional *R*-factors *R* are based on *F*, with *F* set to zero for negative *F*^2^. The threshold expression of *F*^2^ > σ(*F*^2^) is used only for calculating *R*-factors(gt) *etc*. and is not relevant to the choice of reflections for refinement. *R*-factors based on *F*^2^ are statistically about twice as large as those based on *F*, and *R*- factors based on ALL data will be even larger.

### Fractional atomic coordinates and isotropic or equivalent isotropic displacement parameters (Å^2^)

**Table:**

	*x*	*y*	*z*	*U*_iso_*/*U*_eq_	
C1	0.1297 (9)	−0.2248 (9)	0.7468 (4)	0.0382 (19)	
H1	0.0668	−0.2119	0.7912	0.046*	
C2	0.0341 (10)	−0.3449 (9)	0.6867 (4)	0.042 (2)	
H2	−0.0854	−0.4160	0.6918	0.050*	
C3	0.1202 (10)	−0.3567 (9)	0.6187 (4)	0.0378 (18)	
H3	0.0554	−0.4305	0.5761	0.045*	
C4	0.3067 (10)	−0.2560 (9)	0.6148 (4)	0.0334 (17)	
C5	0.4113 (10)	−0.2614 (9)	0.5466 (4)	0.0309 (17)	
C6	0.4200 (11)	−0.3667 (10)	0.4217 (4)	0.047 (2)	
H6	0.3620	−0.4352	0.3767	0.057*	
C7	0.6080 (12)	−0.2729 (10)	0.4227 (4)	0.046 (2)	
H7	0.6723	−0.2817	0.3785	0.056*	
C8	0.6015 (10)	−0.1635 (9)	0.5474 (4)	0.0377 (19)	
C9	0.6942 (9)	−0.0530 (9)	0.6161 (4)	0.0307 (17)	
C10	0.8851 (10)	0.0455 (9)	0.6205 (4)	0.0368 (18)	
H10	0.9584	0.0432	0.5785	0.044*	
C11	0.9615 (10)	0.1464 (9)	0.6892 (4)	0.0383 (19)	
H11	1.0859	0.2162	0.6939	0.046*	
C12	0.8503 (10)	0.1414 (9)	0.7502 (4)	0.0390 (19)	
H12	0.9055	0.2066	0.7963	0.047*	
C13	0.5930 (9)	−0.0467 (8)	0.6809 (4)	0.0306 (17)	
C14	0.3934 (9)	−0.1466 (8)	0.6793 (4)	0.0283 (16)	
C15	0.6273 (9)	0.4436 (8)	0.8808 (3)	0.0243 (15)	
C16	0.7009 (9)	0.6013 (9)	0.9285 (4)	0.0287 (16)	
C17	0.7670 (9)	0.4280 (10)	1.0288 (4)	0.0352 (18)	
H17	0.8113	0.4179	1.0804	0.042*	
C18	0.7034 (9)	0.2695 (9)	0.9810 (4)	0.0314 (17)	
H18	0.7119	0.1568	1.0002	0.038*	
C19	0.5404 (10)	0.4446 (10)	0.7980 (4)	0.0346 (18)	
C20	0.7100 (11)	0.7927 (9)	0.9014 (4)	0.0329 (17)	
N1	0.3064 (8)	−0.1266 (7)	0.7451 (3)	0.0311 (14)	
N2	0.6678 (8)	0.0493 (7)	0.7479 (3)	0.0328 (14)	
N3	0.3191 (8)	−0.3632 (7)	0.4820 (3)	0.0376 (15)	
N4	0.7025 (8)	−0.1691 (8)	0.4849 (3)	0.0386 (15)	
N5	0.6314 (7)	0.2789 (7)	0.9087 (3)	0.0292 (14)	
N6	0.7668 (7)	0.5927 (7)	1.0039 (3)	0.0321 (14)	
O1	0.4150 (7)	0.3020 (6)	0.7750 (2)	0.0389 (12)	
O2	0.5959 (7)	0.5679 (7)	0.7600 (3)	0.0494 (14)	
O1W	0.2264 (8)	0.0860 (8)	0.8973 (3)	0.0411 (14)	
O3	0.5474 (7)	0.8538 (6)	0.8915 (3)	0.0368 (12)	
O4	0.8788 (7)	0.8715 (7)	0.8956 (3)	0.0536 (15)	
Co	0.46559 (14)	0.06529 (13)	0.83165 (5)	0.0332 (3)	
HW1A	0.113 (11)	0.005 (10)	0.905 (4)	0.06 (3)*	
HW1B	0.200 (10)	0.182 (10)	0.910 (4)	0.05 (3)*	

### Atomic displacement parameters (Å^2^)

**Table:**

	*U*^11^	*U*^22^	*U*^33^	*U*^12^	*U*^13^	*U*^23^
C1	0.029 (4)	0.047 (5)	0.037 (5)	−0.004 (4)	0.011 (3)	0.000 (4)
C2	0.040 (4)	0.046 (5)	0.034 (5)	−0.016 (4)	0.010 (4)	−0.001 (4)
C3	0.038 (4)	0.037 (5)	0.034 (5)	−0.001 (4)	−0.004 (4)	−0.006 (4)
C4	0.034 (4)	0.027 (4)	0.038 (4)	−0.001 (3)	0.004 (3)	−0.002 (3)
C5	0.040 (4)	0.032 (4)	0.021 (4)	0.008 (3)	0.001 (3)	0.004 (3)
C6	0.051 (5)	0.064 (6)	0.025 (4)	0.002 (4)	0.009 (4)	−0.008 (4)
C7	0.059 (6)	0.051 (6)	0.032 (5)	0.014 (4)	0.012 (4)	0.001 (4)
C8	0.036 (4)	0.030 (4)	0.053 (5)	0.008 (4)	0.024 (4)	0.011 (4)
C9	0.031 (4)	0.036 (5)	0.024 (4)	0.003 (3)	0.004 (3)	−0.001 (3)
C10	0.032 (4)	0.034 (5)	0.046 (5)	0.006 (3)	0.009 (4)	0.005 (4)
C11	0.032 (4)	0.039 (5)	0.044 (5)	−0.003 (3)	0.016 (4)	0.008 (4)
C12	0.039 (5)	0.038 (5)	0.040 (5)	0.003 (4)	0.002 (4)	0.011 (4)
C13	0.038 (4)	0.022 (4)	0.030 (4)	0.001 (3)	−0.003 (3)	0.000 (3)
C14	0.034 (4)	0.015 (4)	0.034 (4)	−0.001 (3)	0.000 (3)	0.002 (3)
C15	0.030 (4)	0.020 (4)	0.025 (4)	0.007 (3)	0.004 (3)	0.005 (3)
C16	0.025 (4)	0.030 (4)	0.031 (4)	0.001 (3)	0.008 (3)	0.003 (3)
C17	0.039 (4)	0.039 (5)	0.027 (4)	0.004 (4)	0.001 (3)	0.005 (4)
C18	0.033 (4)	0.026 (4)	0.038 (5)	0.004 (3)	0.012 (3)	0.012 (3)
C19	0.039 (4)	0.032 (5)	0.035 (5)	0.006 (4)	0.013 (4)	−0.001 (4)
C20	0.036 (4)	0.030 (5)	0.031 (4)	−0.005 (4)	0.010 (4)	−0.001 (3)
N1	0.030 (3)	0.026 (3)	0.037 (4)	0.002 (3)	0.006 (3)	0.000 (3)
N2	0.035 (3)	0.030 (3)	0.032 (4)	−0.006 (3)	0.009 (3)	0.000 (3)
N3	0.044 (4)	0.038 (4)	0.029 (4)	0.004 (3)	0.003 (3)	0.001 (3)
N4	0.041 (4)	0.045 (4)	0.032 (4)	0.006 (3)	0.013 (3)	0.004 (3)
N5	0.026 (3)	0.035 (4)	0.030 (4)	0.007 (3)	0.009 (3)	0.011 (3)
N6	0.028 (3)	0.029 (4)	0.038 (4)	−0.003 (3)	0.011 (3)	0.002 (3)
O1	0.050 (3)	0.032 (3)	0.030 (3)	−0.003 (2)	−0.003 (2)	0.001 (2)
O2	0.063 (4)	0.041 (4)	0.043 (3)	−0.002 (3)	0.008 (3)	0.007 (3)
O1W	0.035 (3)	0.030 (4)	0.055 (4)	−0.009 (3)	0.015 (3)	−0.009 (3)
O3	0.036 (3)	0.033 (3)	0.045 (3)	0.006 (2)	0.014 (2)	0.009 (2)
O4	0.042 (3)	0.050 (4)	0.064 (4)	−0.017 (3)	0.009 (3)	0.009 (3)
Co	0.0347 (6)	0.0307 (6)	0.0323 (6)	−0.0037 (4)	0.0069 (4)	0.0004 (4)

### Geometric parameters (Å, °)

**Table:**

C1—N1	1.332 (7)	C13—N2	1.345 (8)
C1—C2	1.383 (9)	C13—C14	1.463 (9)
C1—H1	0.9300	C14—N1	1.357 (7)
C2—C3	1.381 (8)	C15—N5	1.351 (7)
C2—H2	0.9300	C15—C16	1.399 (8)
C3—C4	1.403 (9)	C15—C19	1.504 (9)
C3—H3	0.9300	C16—N6	1.353 (8)
C4—C14	1.378 (8)	C16—C20	1.525 (9)
C4—C5	1.453 (9)	C17—N6	1.328 (8)
C5—N3	1.364 (8)	C17—C18	1.393 (8)
C5—C8	1.403 (9)	C17—H17	0.9300
C6—N3	1.318 (8)	C18—N5	1.315 (8)
C6—C7	1.378 (9)	C18—H18	0.9300
C6—H6	0.9300	C19—O2	1.211 (7)
C7—N4	1.345 (8)	C19—O1	1.288 (7)
C7—H7	0.9300	C20—O4	1.242 (7)
C8—N4	1.353 (8)	C20—O3	1.257 (8)
C8—C9	1.446 (9)	Co—N1	2.116 (5)
C9—C13	1.389 (8)	Co—N2	2.124 (5)
C9—C10	1.403 (9)	Co—N5	2.135 (5)
C10—C11	1.389 (9)	Co—O3^i^	2.050 (5)
C10—H10	0.9300	Co—O1W	2.110 (5)
C11—C12	1.370 (8)	Co—O1	2.125 (5)
C11—H11	0.9300	O1W—HW1A	0.95 (7)
C12—N2	1.338 (8)	O1W—HW1B	0.78 (7)
C12—H12	0.9300	O3—Co^ii^	2.050 (5)
			
N1—C1—C2	124.0 (7)	N6—C16—C20	115.1 (6)
N1—C1—H1	118.0	C15—C16—C20	123.7 (6)
C2—C1—H1	118.0	N6—C17—C18	122.7 (6)
C3—C2—C1	118.5 (6)	N6—C17—H17	118.6
C3—C2—H2	120.7	C18—C17—H17	118.6
C1—C2—H2	120.7	N5—C18—C17	120.2 (6)
C2—C3—C4	119.1 (6)	N5—C18—H18	119.9
C2—C3—H3	120.5	C17—C18—H18	119.9
C4—C3—H3	120.5	O2—C19—O1	127.2 (7)
C14—C4—C3	117.7 (6)	O2—C19—C15	120.0 (7)
C14—C4—C5	119.0 (6)	O1—C19—C15	112.7 (6)
C3—C4—C5	123.4 (6)	O4—C20—O3	128.2 (7)
N3—C5—C8	121.2 (6)	O4—C20—C16	115.6 (7)
N3—C5—C4	118.2 (6)	O3—C20—C16	116.1 (6)
C8—C5—C4	120.6 (6)	C1—N1—C14	116.6 (6)
N3—C6—C7	122.6 (7)	C1—N1—Co	127.8 (5)
N3—C6—H6	118.7	C14—N1—Co	115.5 (4)
C7—C6—H6	118.7	C12—N2—C13	116.6 (6)
N4—C7—C6	122.8 (7)	C12—N2—Co	127.5 (5)
N4—C7—H7	118.6	C13—N2—Co	115.3 (4)
C6—C7—H7	118.6	C6—N3—C5	116.3 (6)
N4—C8—C5	121.5 (7)	C7—N4—C8	115.6 (6)
N4—C8—C9	118.3 (6)	C18—N5—C15	119.2 (6)
C5—C8—C9	120.2 (6)	C18—N5—Co	127.6 (5)
C13—C9—C10	118.4 (6)	C15—N5—Co	112.2 (4)
C13—C9—C8	119.1 (6)	C17—N6—C16	116.7 (6)
C10—C9—C8	122.6 (6)	C19—O1—Co	114.9 (4)
C11—C10—C9	118.1 (6)	Co—O1W—HW1A	134 (4)
C11—C10—H10	121.0	Co—O1W—HW1B	120 (6)
C9—C10—H10	121.0	HW1A—O1W—HW1B	104 (7)
C12—C11—C10	118.9 (7)	C20—O3—Co^ii^	131.4 (4)
C12—C11—H11	120.6	O3^i^—Co—O1W	91.3 (2)
C10—C11—H11	120.6	O3^i^—Co—N1	88.77 (19)
N2—C12—C11	124.5 (7)	O1W—Co—N1	96.0 (2)
N2—C12—H12	117.7	O3^i^—Co—N2	96.21 (19)
C11—C12—H12	117.7	O1W—Co—N2	169.4 (2)
N2—C13—C9	123.6 (6)	N1—Co—N2	76.8 (2)
N2—C13—C14	115.9 (6)	O3^i^—Co—O1	173.06 (19)
C9—C13—C14	120.5 (6)	O1W—Co—O1	91.9 (2)
N1—C14—C4	123.9 (6)	N1—Co—O1	96.98 (19)
N1—C14—C13	115.5 (6)	N2—Co—O1	81.41 (19)
C4—C14—C13	120.6 (6)	O3^i^—Co—N5	96.8 (2)
N5—C15—C16	119.9 (6)	O1W—Co—N5	87.2 (2)
N5—C15—C19	116.4 (6)	N1—Co—N5	173.5 (2)
C16—C15—C19	123.7 (6)	N2—Co—N5	99.2 (2)
N6—C16—C15	121.2 (6)	O1—Co—N5	77.22 (19)

Symmetry codes: (i) *x*, *y*−1, *z*; (ii) *x*, *y*+1, *z*.

### Hydrogen-bond geometry (Å, °)

**Table:**

*D*—H···*A*	*D*—H	H···*A*	*D*···*A*	*D*—H···*A*
O1W—HW1A···O4^iii^	0.95 (7)	1.76 (7)	2.680 (7)	164 (6)
O1W—HW1B···N6^iv^	0.78 (7)	2.15 (7)	2.851 (8)	149 (7)

Symmetry codes: (iii) *x*−1, *y*−1, *z*; (iv) −*x*+1, −*y*+1, −*z*+2.
